# Single molecule conformational analysis of DNA G-quadruplexes

**DOI:** 10.1016/j.biochi.2008.01.015

**Published:** 2008-08

**Authors:** Pravin S. Shirude, Shankar Balasubramanian

**Affiliations:** Department of Chemistry, University of Cambridge, Lensfield Road, Cambridge, CB2 1EW, UK

**Keywords:** DNA, G-quadruplex, Single molecule FRET, Conformations, hTelo

## Abstract

Single molecule fluorescence resonance energy transfer (FRET) can be employed to study conformational heterogeneity and real-time dynamics of biological macromolecules. Here we present single molecule studies on human genomic DNA G-quadruplex sequences that occur in the telomeres and in the promoter of a proto-oncogene. The findings are discussed with respect to the proposed biological function(s) of such motifs in living cells.

## 1 Introduction

DNA is a dynamic molecule whose canonical double-helix conformation must be disrupted to assume alternative structural forms to fulfil certain functions in nature. Sequence motifs containing stretches of tandem Gs can form four-stranded structures called G-quadruplexes that comprise stacked tetrads of mutually hydrogen-bonded guanines [1,2]. G-quadruplex motifs occur throughout the human genome [3], raising the possibility of associated function. The single-stranded G-rich overhang of telomeres in various species can form G-quadruplexes [4–6] and their formation in ciliates is regulated by telomere binding proteins [7]. Quadruplex motifs are also prevalent in non-telomeric parts of the genome [3], particularly in gene promoters [8] that include *c-MYC* [9,10], KRAS [11], VEGF [12], c-kit [13,14] and BCL-2 [15]. These quadruplex structures may act as topological switches that are coupled to the initiation of transcription. If quadruplex structures are to form in such duplex regions they must necessarily compete with the DNA duplex, despite high thermal stability normally associated with G–C rich duplex. Single molecule fluorescence resonance energy transfer (FRET) can be employed to directly study the conformational analysis of such G-quadruplex forming sequences.

Single-molecule detection is a powerful approach for directly analysing biomolecular properties without the temporal and population averaging that results in conventional ensemble studies. Often, conformational fluctuations are difficult to detect using ensemble studies [16–18]. FRET is a spectroscopic technique sensitive to distances and changes in distance, in the 10–80 Å range [19,20]. To apply FRET, the biomolecule(s) under investigation must be labelled with a donor and an acceptor fluorophore. An excited donor can transfer its energy to the acceptor via an induced-dipole, induced-dipole interaction, whereby the efficiency of energy transfer, *E*, is given by [1 + (*R*/*R*_0_)^6^]^−1^, where *R* is the distance between the donor and acceptor. *R*_0_ is the distance at which 50% of the energy is transferred and it can be calculated as described in Eq. (1.0)

(1.0) $$R_{0}=9.79\times 10^{3}\sqrt[{6}]{J\varphi^{\text{D}}\kappa^{2}n^{-4}}$$

where *J* is the overlap integral, *φ*^D^ is the quantum yield of the donor in the absence of the acceptor, *k*^2^ is the orientation factor in space between the transition dipoles for donor and acceptor, and *n* is the refractive index of the surrounding medium, usually assumed to be equal to that of the surrounding bulk medium [19]. Changes in the distance between the two labelled sites of a biological molecule can result in a measurable change in *E*. Therefore, structural changes within biological molecules or relative dynamics between two different molecules can be detected via FRET changes [21]. Two general formats employed for single molecule FRET measurements are confocal detection and excitation with an evanescent wave generated by total internal reflection. Solution phase experiments involve the analysis of donor and acceptor photon bursts as the subject molecule diffuses through the confocal probe volume. While this format provides a solution environment, the diffusion limited temporal window (∼ms) precludes the investigation of slow (>10 ms) dynamics. The total internal reflection microscope allows a whole field of molecules, typically several hundred of them, to be detected simultaneously enabling favourable statistics for the measurements. However, wide-field detection requires the use of cameras, which limit the data acquisition rate to 1 kilocycle per second or slower using state-of-the-art charge couple device (CCD) cameras with on-chip amplification. Higher data acquisition rates, up to tens of megacycles per second, can be achieved with confocal microscopes using sensitive point detectors, such as avalanche photodiodes or photomultipliers. The single molecule detection of molecules immobilised at a surface provides an extended observation window limited only by photobleaching, but requires care to avoid perturbation of biomolecules that may be induced by surface interactions. This perturbation has been rather insignificant for most of the DNA/RNA systems studied to date, at neutral or basic pH values. For systems that involve proteins, which tend to interact strongly with surfaces through hydrophobic interactions, surface passivation is often required to retain the integrity of the system. Polyethylene glycol (PEG) is a commonly used passivating reagent [22]. Another method is to entrap the biomolecule in a lipid vesicle attached to a surface [23].

**Fig. 1:**
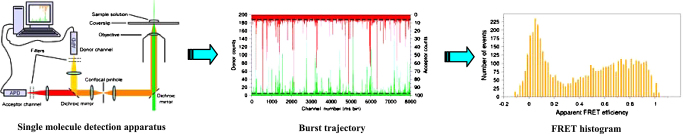
Schematic of single molecule FRET analysis.

The efficiency of energy transfer can be measured by determining the donor lifetime or its fluorescence intensity in the presence and absence of the acceptor. Alternatively, it can be obtained by calculating the ratio between the acceptor fluorescence intensity and the total intensity (donor plus acceptor). With a confocal microscope, the raw data collected from a single molecule experiment is known as a burst trajectory, an example of which is shown in Fig. 1. There are two trajectories: one for the donor and one for the acceptor. After corrections for leakage of donor fluorescence into the acceptor channel and vice versa, the remaining signals represent the intensities of the donor and acceptor, *I*_d_ and *I*_a_ respectively. The FRET efficiency (*E*) can then be calculated using Eq. (1.1), where *γ* is a term including information about the quantum yields of the donor and acceptor and the collection efficiencies of the detectors used to measure their intensities. A distribution of quantum yields is likely to occur in individual molecules, therefore rather than assuming that each molecule has an identical quantum yield, in single molecule studies *γ* is assumed to be equal to one [24–25] and it is the *apparent* FRET efficiency, *E*_app_, that is calculated (Eq. (1.2)).

(1.1) $$E=I_{\text{a}}/\left(I_{\text{a}}+\gamma I_{\text{d}}\right)$$

(1.2) $$E_{\mathrm{app}}=I_{\text{a}}/\left(I_{\text{a}}+I_{\text{d}}\right)$$

One normally collects donor and acceptor burst intensities for a large number of events. By plotting a histogram of *E* vs. number of events, one can observe the distribution of conformational species in the solution.

Single molecule FRET [26–29] opens up new opportunities to probe the real time structural changes of biological molecules. Since the first demonstration of single molecule FRET [30] there have been a number of studies on a wide range of bio-molecular systems that include: the oligomerization of membrane proteins in a living cell [31,32]; protein folding [33–37]; protein conformational changes [38–44]; RNA folding and catalysis [45–55]; DNA structural dynamics [25,55–60]; and DNA-protein interactions [61–64]. FRET has been applied at the ensemble level to study DNA quadruplex formation and unfolding [65–71]. In the single molecule regime, it has been possible to gain further insights into the distribution of G-quadruplex conformations and study how these vary with changing conditions. Here we describe single molecule FRET studies on the human telomeric intramolecular quadruplex (htelo) and on a G-quadruplex motif found within the promoter of the proto-oncogene (c-kit) [57,58,82].

## 2 The human telomere G-quadruplex

**Fig. 2:**
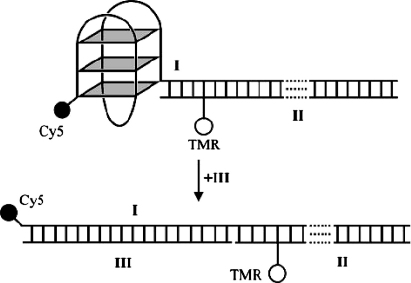
Unfolding of the quadruplex system **I**:**II** in the presence of **III**. Copyright (2003) National Academy of Sciences of the United States of America [57].

Telomerase function and telomere maintenance are critical for the division of cancer cells. Thus the human telomeric G-quadruplex (htelo) [d(GGG TTA GGG TTA GGG TTA GGG)] has been the subject of many studies and is being considered as a potential molecular target for the development of novel anticancer agents [72,73]. Detailed structural studies had initially provided evidence for two distinct folds for htelo in the presence of either sodium or potassium ions [74,75]. An NMR spectroscopic study, where the dominant monovalent cation was sodium, showed that htelo exists predominantly with an antiparallel arrangement of strands with a diagonal loop across a terminal tetrad and edgewise loops [74]. In contrast, an X-ray crystal structure of the same oligonucleotide, in the presence of potassium, showed all four strands to be parallel with all loops located down the side of the quadruplex, leaving both terminal tetrads exposed [75]. More recently, additional conformations called mixed hybrid structures of the human telomeric intramolecular G-quadruplex [76,77] have been observed by NMR spectroscopy. Ensemble techniques can often fail to resolve the coexistence of more than one structure. Ying et al. [57] explored the possibility of distinct conformations of htelo by single molecule FRET. The dual-labelled quadruplex system employed **I**:**II** is shown in Fig. 2. The sequences of these oligonucleotides are as follows: **I**, 5′-Cy5-GGG TTA GGG TTA GGG TTA GGG AGA GGT AAA AGG ATA ATG GCC ACG GTGCGGACG GC-3′; which contains the human telomeric repeat motif d(GGG TTA GGG TTA GGG TTA GGG), which can form an intramolecular G quadruplex, with Cy5 coupled to the 5′ terminus by phosphoramidite methodology; **II**, 5′-GCC GTC CGC ACC GTG GCC ATT ATC CTT *TTA CCT CT-3′ (*T represents the TAMRA-dT residue) is the complement of the 35-nucleotide overhang of **I**, with tetramethylrhodamine (TMR) coupled to a thymine (T28) by a six-carbon linkage; **III**, 5′-CCC TAA CCC TAA CCC TAA CCC-3′ is the complement of the quadruplex-forming region of **I**.

The quadruplex is based on the htelo sequence and is connected to a 35-bp duplex. The overall design places the two dyes about 47 Å apart in the folded state. This value was close to the estimated Forster distance of 53 Å [33], which enhances the sensitivity of FRET changes caused by alterations in structure. Furthermore, positioning of the TMR dye in the duplex minimizes short-range quenching interactions with Cy5 or guanines in the quadruplex [66,78]. Unfolding the quadruplex of **I**:**II** in the presence of a complementary strand leads to a large decrease in FRET efficiency (from ∼70% to ∼5%) as the concomitant distance between the two fluorophores increases.

### 2.1 Structural heterogeneity and thermodynamics

**Fig. 3:**
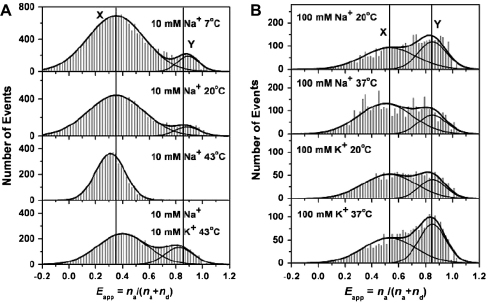
Single molecule FRET histograms for HTIQ. (A) From top to bottom, all in 10 mM NaCl and 10 mM Tris–HCl (pH 7.4), at 7 °C, 20 °C, 43 °C, and 43 °C with additional 10 mM KCl. (B) From top to bottom, all in 10 mM Tris–HCl (pH 7.4), at 20 °C, 100 mM NaCl; at 37 °C, 100 mM NaCl; at 20 °C 100 mM KCl; and at 37 °C, 100 mM KCl. Solid curves are the best fit to guassian functions. The “zero” peaks, largely due to inactive Cy5, have been subtracted for clarity. Copyright (2003) National Academy of Sciences of the United States of America [57].

Single-molecule FRET measurements on the system **I**:**II** showed the presence of two clearly resolved species in the presence of both K^+^ and Na^+^ metal ions. It was observed that the equilibrium between the two subpopulations could be shifted by changes in temperature and also by changing the monovalent metal ions present. In the presence of low salt concentration (10 mM Na^+^ or K^+^), the low-FRET species (X) was centred at 0.35, while the high-FRET species (Y) was centred at 0.85 (Fig. 3). At high salt concentration (100 mM Na^+^ or K^+^), the mean apparent FRET efficiency of X was increased from 0.35 to 0.52, while the mean FRET efficiency of Y was unaffected by the change of salt concentration. It was proposed that the two structures (X and Y) may be due to the parallel and antiparallel folds of the same quadruplex based on the previously defined structures [74,75]. Structural assignments of the two subpopulations were proposed on the basis of energy minimized modelling of the antiparallel and parallel structures [57]. Assuming a Forster distance, *R*_0_, of 7.1 nm for the TMR/Cy5 pair, and an estimated contribution of the linkers to the interfluorophore distance, it was proposed that X corresponds to the antiparallel quadruplex, and that Y corresponds to the parallel quadruplex (The measured quantum yield of TMR in this system was 0.81, leading to a calculated Forster radius, *R*_0_, of 7.1 nm, assuming an orientational factor, *k*^2^, of 2/3. This value is higher than commonly measured for the TMR/Cy5 pair, largely because of the high quantum yield of TMR in this system). The free energy difference between these structures was small (<1 kcal mol^−1^) over the temperature range investigated. The two forms did not interconvert during the time a molecule spends in the excitation volume of a confocal microscope (∼1 ms), and both conformations were unfolded and trapped as duplex structures within minutes by means of hybridization to a complementary strand, suggesting that the dynamic interconversion between different conformations may occur in the time window between 1 ms and a few minutes.

**Fig. 4:**
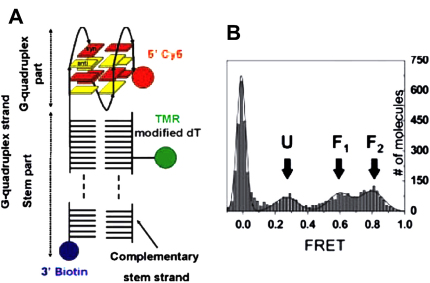
Conformational switching of human telomeric DNA with FRET. (A) Schematic of DNA construct employed. (B) Expanded view of FRET histogram taken at 2 mM K^+^ showing three nonzero FRET peaks, assigned U (unfolded), F1, and F2 (folded). Also shown is the fit by four Gaussians. Copyright (2005) National Academy of Sciences of the United States of America [58].

To probe the dynamic properties of the telomeric DNA, Lee et al. subsequently [58] performed single-molecule FRET experiments on a similar construct to **I**:**II** (Fig. 2) that comprised a terminal biotin for immobilization to a streptavidin coated quartz surface to extend the observation time (Fig. 4A). At 2 mM K^+^, three species were observed (Fig. 4B), one at a high FRET efficiency (∼0.8), another at an intermediate FRET efficiency (∼0.6), and the third at a low FRET efficiency (∼0.3). As K^+^ concentration was increased, the population of the low FRET state decreased and the higher FRET states increased. Since the increased K^+^ concentration stabilizes the G-quadruplex structure, the low FRET state was considered to be an unfolded conformation (U) (i.e. disordered single stranded overhang) and the other two states to folded conformations (G-quadruplex structures, F1 and F2) (Fig. 4B). This difference in the profile of FRET histogram as compared to previously reported by Ying et al. [57] was a result of differences in the resolution of the two instruments used for the respective studies, whereby the instrument used by Lee et al. had better signal to noise ratio and was able to better resolve the ‘unfolded’ peak. It was also shown that for a mutated sequence in which a guanine in the middle quartet was replaced by a thymine, G-quadruplex formation was significantly disrupted. In this study it was apparent that the subpopulation U, F1 and F2 were interconverting in support of the proposal by Ying et al [57].

### 2.2 Kinetics and dynamics

**Fig. 5:**
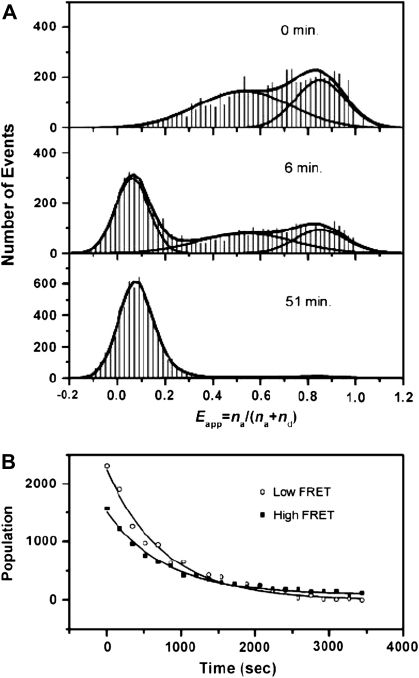
(A) Changes in single-molecule FRET histograms during the opening of the DNA quadruplex by hybridization to 7 nM **III**. The ‘zero’ peak at *t* = 0 has been subtracted from all histograms for clarity. The experiment was carried out at 20 °C in 100 mM NaCl and 10 mM Tris–HCl (pH 7.4). (B) Kinetic traces for the low- and high-FRET subpopulations. Data were fit to a single exponential. Copyright (2003) National Academy of Sciences of the United States of America [57].

**Fig. 6:**
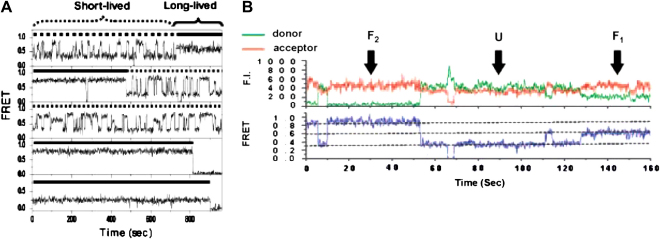
Conformational switching of human telomeric DNA with FRET. (A) Extreme conformational diversity revealed by single-molecule dynamics: representative FRET time traces of single molecules at 2 mM K^+^ and room temperature (0.9 s integration time). (B) Time traces (0.1 s integration time) of donor and acceptor intensities and corresponding FRET from a single molecule at 2 mM K^+^ exhibit interconversion between U, F1, and F2. A transition to zero FRET at 65 s is due to Cy5 blinking. Note that the transition from F2 to F1 occurred through U. Copyright (2005) National Academy of Sciences of the United States of America [58].

Ying et al. demonstrated [57] that two conformations X and Y (Fig. 3) apparently unfold at the same rate in a second-order hybridization reaction (Fig. 5). In the presence of potassium, the activation free energy of unfolding (*ΔG*_activation_ = 22.6 kcal mol^−1^ at 37 °C) was largely entropic (Δ*H*_activation_ = 6.4 ± 0.4 kcal mol^−1^, Δ*S*_activation_ = −52.3 ± 1.4 cal mol^−1^ K^−1^), suggesting that the transition state is pre-organized in a favourable conformation for hybridization to occur. However, in the presence of sodium ions, although the free energy of activation (*ΔG*_activation_ = 22.3 kcal mol^−1^ at 37 °C) was similar, a relatively larger enthalpic contribution was observed (Δ*H*_activation_ = 14.9 ± 0.2 kcal mol^−1^, Δ*S*_activation_ = −23.0 ± 0.8 cal mol^−1^ K^−1^). Therefore it was hypothesized that different transition-state structures exist depending on the presence of either sodium or potassium ions. The analysis of single-molecule dynamics by Lee et al. [58] revealed transitions between all three FRET states (U, F1, and F2), most frequently in 2 mM K^+^ (Fig. 6B). Most of the transitions between F1 and F2 passed through U. A direct transition between two folded conformations without unfolding is highly unlikely considering the folded topology of G-quadruplexes. According to the analysis of dwell times, they further classified the molecular conformations into either long-lived species, if the dwell time was >100 s and short-lived species if the dwell time was <100 s. In total, six distinct states were observed: long-lived states LU, LF1, and LF2, and short-lived states SU, SF1, and SF2 (Fig. 6A). The long-lived folded states, LF1 and LF2 (Fig. 6A), were dominant in high K^+^ concentrations and were assigned to the parallel and antiparallel structures respectively, based on comparison with NMR spectroscopic studies of temperature dependence [79]. The long-lived unfolded state, LU, was highly populated at low K^+^ concentrations and displayed the characteristic salt dependence of a disordered ssDNA. SF1 and SF2 were much less stable than their long-lived counterparts. SU was clearly distinct from LU in having a much shorter lifetime. Although SF1 and SF2 differed from LF1 and LF2 in microscopic detail, their similar FRET values suggested that they may have the same global folds.

### 2.3 Summary

**Fig. 7:**
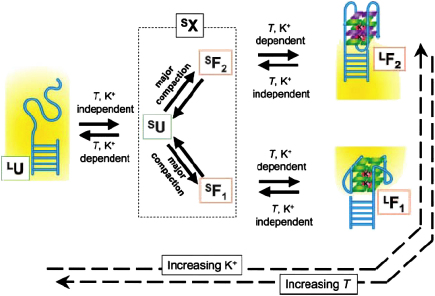
Proposed model for conformational identities and reaction pathways. The cartoon for LU denotes a disordered conformation of the ssDNA, and LF1 and LF2 are depicted as the parallel and antiparallel G-quadruplex structures, respectively, coordinating K^+^ ions between G-quartets. The short-lived species (SX) include SU, SF1, and SF2. The solid arrows represent the proposed reaction paths and the dashed arrows point toward the states that are favoured upon increasing K^+^ concentration or temperature. Copyright (2005) National Academy of Sciences of the United States of America [58].

These studies revealed the conformational heterogeneity of htelo and suggested at least two stable folded conformations, in both sodium- and potassium-containing buffers. The two folded states showed relatively small differences in free energies. The dynamics switching between the quadruplex conformations was explored [58] and a kinetics model was proposed for the interconversion of six distinct states (Fig. 7). Subsequent to the single molecule studies, additional mixed hybrid, and mixed parallel and antiparallel structures of the human telomeric intramolecular G-quadruplex [76-77] have been observed by NMR spectroscopy. We can not rule out the possibility that subpopulations observed in the FRET histograms are in part comprised of mixed hybrid type conformations. Furthermore such additional conformations may help to explain the six states of htelo revealed by the single molecule studies.

## 3 The c-kit -promoter G-quadruplex

The c-kit gene encodes a receptor tyrosine kinase, whose engagement by its ligand triggers signals leading to cell proliferation. c-kit activity is elevated in gastrointestinal stromal tumors (GISTs), and its therapeutic inhibition by small molecules, such as imatinib, is clinically validated. Transcriptional regulation of c-kit expression is complex, involving several activators and repressors [80,81]. Two G-quadruplex sequence motifs have been identified by us within the c-kit promoter region. One c-kit G-quadruplex motif is positioned between −87 and −109 bp upstream of the transcription start site and was shown to fold into a quadruplex in vitro [13]. The other c-kit quadruplex motif is positioned between −140 and −160 bp upstream of the transcription initiation site, occupies a region required for core promoter activity, and was also shown to form a G-quadruplex by nuclear magnetic resonance (NMR), circular dichroism (CD), and ultraviolet (UV) spectroscopic methods [14]. This quadruplex-forming sequence was also shown to have a high level of sequence conservation across human, mouse, rat, and chimpanzee.

**Fig. 8:**
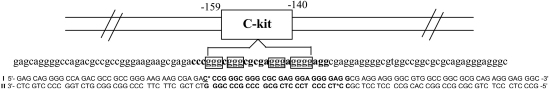
DNA construct −102 to −197 bp upstream of the c-kit transcription start site comprising G repeats that form an intramolecular quadruplex (boxed gs) found in the sequence situated from −140 to −159 bp upstream, the bases with asterisks are attached to fluorophorores through a C6 linker. Reprinted with permission from [82]. Copyright (2007) American Chemical Society.

**Fig. 9:**
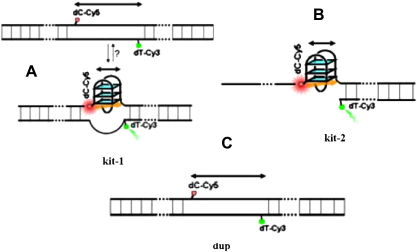
c-kit Intramolecular G-quadruplex system. (A) Duplex to quadruplex interconversion (kit-1). (B) Single stranded c-kit intramolecular quadruplex system (kit-2). (C) Control duplex system that can not form G-quadruplex (dup). Reprinted with permission from [82]. Copyright (2007) American Chemical Society.

In order to explore the conformation of this conserved c-kit G-quadruplex motif we designed a dual-labelled double-stranded system (Fig. 8) [82] in which the c-kit quadruplex motif and flanking sequence elements were from the native human c-kit promoter sequence situated from −102 to −197 bp upstream of the transcription initiation site. The sequence from −140 to −159 bp contains the conserved c-kit quadruplex motif [14], and donor (Cy3) and acceptor (Cy5) fluorophores were incorporated on opposite strands at sites that flanked the G-quadruplex motif. G-quadruplex formation would reduce the separation of the dyes leading to an increase in FRET. The 38 nucleotide double-stranded DNA either side of the quadruplex motif ensures that the two strands in the system do not dissociate and may also introduce constraints that reflect the native sequence context of this quadruplex motif (kit-1, Fig. 9A). We had also designed a similar dual-labelled system comprising 96 nucleotides but lacking a complementary strand opposite to the quadruplex motif (kit-2, Fig. 9B) to address the relevance of the complementary strand on quadruplex conformation. As a control we had also studied a dual-labelled control duplex of same length but lacking a quadruplex forming sequence (Fig. 9C).

**Fig. 10:**
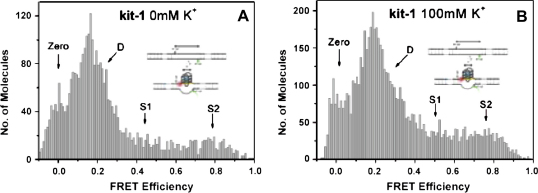
Single-molecule histograms of FRET efficiencies for the kit-1 system: (A) in 0 mM KCl; (B) in 100 mM KCl. All are in 10 mM sodium cacodylate (pH 7.4) at 20 °C. The “zero” peak is largely due to Cy5 dark state. Reprinted with permission from [82]. Copyright (2007) American Chemical Society.

**Fig. 11:**
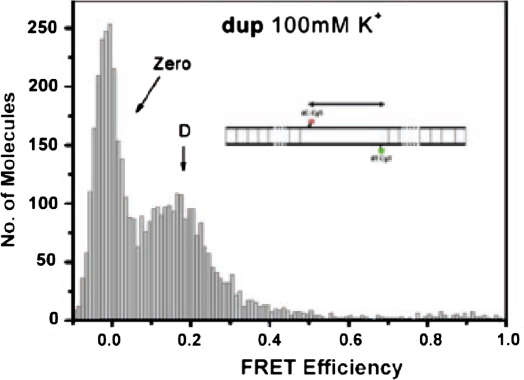
Immobilized single-molecule histograms of apparent FRET efficiencies for DNA duplex (dup) in 100 mM KCl and 10 mM sodium cacodylate (pH 7.4) at 20 °C. The “zero” peak is largely due to Cy5 dark state. Reprinted with permission from [82]. Copyright (2007) American Chemical Society.

Single-molecule FRET measurements were carried out either in free solution, using confocal microscopy [29], or by vesicle encapsulation of single DNA molecules that could then be immobilized [83]. It was shown that the single-molecule analysis either of freely diffusing kit-1 or of immobilized kit-1 both gave comparable data that revealed three subpopulations in the FRET histogram (Fig. 10) [82]. The zero FRET species does not represent a conformational state of the DNA but it was caused by either dark states of the Cy5 acceptor or by incompletely labelled molecules in which Cy5 is absent [84]. The low FRET species (D) was identified as a duplex state of kit-1, consistent with the single FRET population observed for the control duplex dup (Fig. 11). The high FRET subpopulation (S2) was consistent with quadruplex formation [57,58], while the medium FRET structure (S1) was identified as an additional conformation which may be due to partially folded structure. It was shown that all these three subpopulations were observable in the absence of added K^+^ (Fig. 10A). While the population of the high FRET species was slightly increased in 100 mM K^+^ from 23% to 30%. This suggested only a moderate dependence of quadruplex formation on K^+^.

**Fig. 12:**
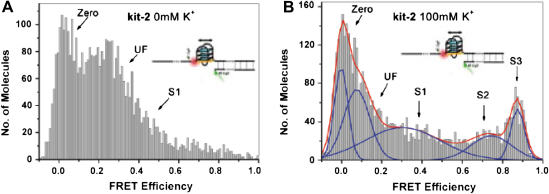
Single-molecule histograms of FRET efficiencies for the kit-2 system: (A) in 0 mM KCl, (B) in 100 mM KCl, with a Gaussian fit to show subpopulations. All are in 10 mM sodium cacodylate (pH 7.4) at 20 °C. The “zero” peak is largely due to Cy5 dark state. Reprinted with permission from [82]. Copyright (2007) American Chemical Society.

Similar single-molecule studies on kit-2, which lacks a DNA strand complementary to the G-quadruplex motif, showed greater structural heterogeneity than kit-1 with three folded structures and one unfolded structure in the presence of 100 mM K^+^ (Fig. 12). In the presence of 100 mM K^+^, the folded conformations were comprised of two high FRET populations (S2 and S3), while one population was at an intermediate FRET efficiency (S1) and the other was at a low FRET efficiency (UF) [82]. We hypothesized that the two high FRET species (S2 and S3) were due to two distinct quadruplex structures, while the medium FRET species (S1) may have been due to partially folded structure and the low FRET species was due to unfolded structures (UF). There were distinctions between kit-1 and kit-2 suggested by the data. First, the single-stranded quadruplex system kit-2 adopts a different profile of structures as compared to the same sequence motif in the presence of its complementary strand (i.e., kit-1). Second, the positive influence of K^+^ on quadruplex formation was more pronounced in the absence of the complementary strand and flanking duplex (kit-2) than in the presence of a native duplex environment (kit-1). The control duplex (dup) (Fig. 11) did not show any structural dependence on K^+^. Studies on freely diffusing kit-1 showed similar FRET values without any dynamic changes during the time a molecule spends in the excitation volume of a confocal microscope (<1 ms). Single-molecule fluorescence time trajectories on immobilized vesicle encapsulated kit-1 revealed that <1% of the molecules showed dynamic fluctuations over an observation time of ∼30 min (data not shown), suggesting that duplex-quadruplex interconversion was a relatively rare event under the conditions employed. For kit-2 it was also observed that <1% of the molecules show dynamic fluctuations in the same time window (data not shown). These studies demonstrated that the c-kit quadruplex motif was able to fold into non-duplex states within a natural extended DNA duplex. There were apparent differences in putative quadruplex structures and the K^+^ dependence on quadruplex formation that appear to result when studying this quadruplex motif in natural duplex, as opposed to the single-stranded form [82].

### 3.1 Summary

The studies on the c-kit promoter quadruplex motif demonstrated that it was able to fold into non-duplex states within a natural extended DNA duplex. There were noticeable differences in putative quadruplex structures and the K^+^ dependence on quadruplex formation that appear to result when studying this quadruplex motif in natural duplex, as opposed to the single-stranded form. The rarity of dynamic fluctuation in the c-kit quadruplex, even in single-stranded form, contrasts with the dynamics observed for the human intramolecular quadruplex. The dynamic behaviour of intramolecular DNA quadruplexes is likely to be an intrinsic property of each sequence and may thus vary significantly between different quadruplexes.

## 4 Overall conclusions and biological implications

The single molecule studies we have described suggest that c-kit and htelo intramolecular DNA G-quadruplexes are conformationally heterogeneous. For the case of htelo this is also supported by several conformations that have been observed by X-ray crystallography and by NMR spectroscopy. The complex dynamic interconversions of htelo between distinct FRET states, on a time scale of seconds to minutes, suggest that any one, or indeed several, of the conformational states may be present at the telomeres in the cells. Other components of the telosome (e.g. proteins) may ultimately govern which conformational state prevails in cells and at what stage during the cell's cycle. The observation of folded structures in the FRET histogram of the c-kit promoter duplex supports that such promoter G-quadruplex motifs can offer an alternative to the duplex structure. This is consistent with reports that link G-quadruplex motifs to regions that show hypersensitivity of chromosal DNA towards single-stranded nucleases, since nuclease hypersensitivity is associated with non-duplex DNA structures. Furthermore it challenges the dogma that G,C-rich DNA necessarily forms a very stable duplex. If indeed such motifs have natural function, these studies would support the view that the molecular mechanism involves changes in the topology of the DNA.

Single molecule FRET has proven to be a valuable biophysical approach to study two distinct genomic G-quadruplexes. The method complements classical structural and biophysical approaches and further studies on other genomic G-quadruplex motifs will help us to establish whether there are generalizations that can be drawn about the associated conformational dynamics.
